# What is an ‘ideally imperfect’ crystal? Is kinematical theory appropriate?

**DOI:** 10.1107/S2053273315018975

**Published:** 2016-01-01

**Authors:** Paul F. Fewster

**Affiliations:** a Panalytical Research, Sussex Innovation Centre, Science Park Square, Brighton, East Sussex BN1 9SB, England

**Keywords:** imperfect crystals, kinematical theory, dynamical theory

## Abstract

Most materials are crystalline because atoms and molecules tend to form ordered arrangements, and since the interatomic distances are comparable with the wavelength of X-rays, their interaction creates diffraction patterns. The intensity in these patterns changes with crystal quality. Perfect crystals, *e.g*. semiconductors, fit well to dynamical theory, whereas crystals that reveal the stereochemistry of complex biological molecules, the structure of organic and inorganic molecules and powders are required to be fragmented (termed ‘ideally imperfect’) to justify the use of the simpler kinematical theory. New experimental results of perfect and imperfect crystals are interpreted with a fundamental description of diffraction, which does not need fragmented crystals but just ubiquitous defects. The distribution of the intensity is modified and can influence the interpretation of the patterns.

## Abstract 2

The diffraction from imperfect crystals and the applicability of kinematical theory are described.

## 1. Introduction   

The kinematical theory is used widely in X-ray diffraction and assumes that the amplitude scattered from each plane is additive, such that the intensity *I_hkl_* ∝ |*F_hkl_*|^2^. Experimentally the intensities are determined from the scattered X-rays in the vicinity of the peaks. There is a problem with this explanation as pointed out by Darwin (1914 ▸) in that the scattered beam will be incident at an angle close to the condition for strong scattering from the underside of the planes above, creating a re-scattered beam which will interfere with the incident beam travelling in the same direction. This not only reduces the intensity of the incident beam but also weakens the scattered beam. This effect is described with dynamical theory, Ewald (1916 ▸, 1917 ▸), which leads to a complex relationship between intensity and the structure factor, making it unsuitable for routine structure determination. Dynamical theory results in very close fits to the structure of perfect crystals, *e.g.* semiconductor wafers, whereas kinematical theory seems to work for biological and chemical structures.

Darwin (1922 ▸) proposed that crystals are in general imperfect, and could be considered as a conglomeration of small perfect crystal blocks. These have to be sufficiently small for the scattered beams to be weak and thin enough to limit the re-scattering impact on the incident beam. This implies, as Authier (2001 ▸) describes, that the scattered amplitude from each crystal block has no phase relationship with its neighbours such that the overall intensity is the sum of the intensity from each block. Therefore the width of the diffraction peak will be associated with the crystal blocks and not the crystal as a whole. This description defines the crystal microstructure rather precisely, regions small enough to avoid dynamical effects, with orientations that are still small *etc*.

Since dynamical effects are unavoidable in the Bragg description is there an alternative explanation, without putting unreasonable demands on the crystal microstructure? Or is the sample description a convenient description to make the theory fit?

## 2. Observation of the intensity from nearly perfect and imperfect crystals   

**Figure 1:**
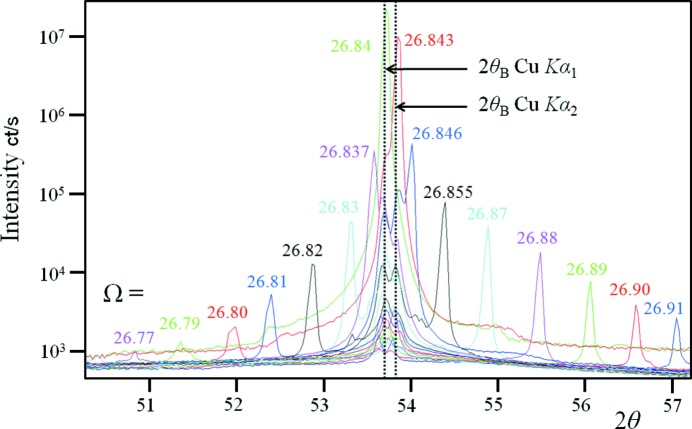
The experimental demonstration of the enhancement at the scattering angle 2θ_B_, for various angles of incidence Ω to the crystal planes. The sharp peaks are the specular peaks and the central double peaks are the enhancement peaks. The measurements were collected in 20 s with the detector (PIXcel 3D) centred on the 113 scattering angle for the Bragg condition using an X-ray mirror, with a beam size in the scattering plane of 1.2 mm.

The diffraction profile from a set of planes from a perfect crystal is predicted to reveal two peaks, one associated with a mirror reflection and the other associated with the spacing between the planes (Fewster, 2014 ▸). The position of the mirror reflection varies as the crystal is rotated, whereas the latter is stationary. This can be observed in Fig. 1 ▸ for the (113) crystal planes of a high-quality Ge (001) orientated wafer. The (113) crystal plane, inclined at φ = 25.3° to the surface, avoids any interference from surface reflections. For this set of crystal planes and Cu *K*α wavelength, the incident angle to the crystal planes will satisfy the Bragg condition when Ω = 2θ_B_/2 where 2θ_B_ ∼ 53.68°. The profiles in Fig. 1 ▸ were obtained by setting Ω at various angles close to 2θ_B_/2 and the intensity is captured on a 7° position-sensitive detector centred on 2θ_B_ (Fewster, 2015 ▸). For each setting there is a sharp specular peak and a double-peaked profile at the scattering angle 2θ_B_. The sharp mirror peak reflects all wavelengths in the same direction and is purely a function of the incident angle. The two central peaks correspond to the 2θ_B_ angles for the Cu *K*α_1_ and Cu *K*α_2_ wavelengths, *i.e.* they appear at the same position for all incident angles. As the specular peak approaches 2θ_B_ for one of the wavelengths the intensity increases, the peaks merge and the Bragg condition is satisfied for that combination of λ and *d*. Away from the traditional Bragg condition (Ω = 2θ_B_/2) the intensities of the peaks are low but they are nonetheless clearly present.

**Figure 2:**
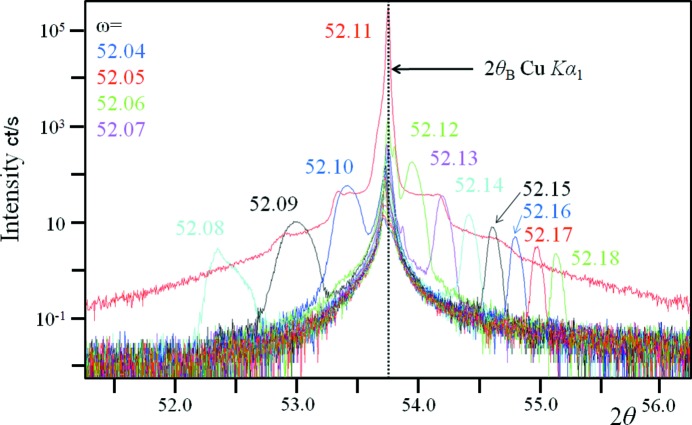
The profiles close to the scattering from the (113) planes from an imperfect (001) gallium arsenide wafer at several orientations in ω, using the same geometry as Fig. 1 ▸, except that a 4× channel-cut crystal was used to isolate a single wavelength and create a narrow incident beam. The beam size in the scattering plane is 0.3 mm. The undulations of the crystal planes are revealed by the shape of the specular profiles at each ω. The strain variation is emphasized by the broad enhancement peaks. When the specular beam is below the critical angle, it cannot emerge from the crystal, *i.e.* ω < 52.07° but the enhancement peak is still present.

In the case of an imperfect crystal, the diffraction profile is more complicated (Fig. 2 ▸). The mirror reflection is no longer sharp but broad, the 2θ_B_ peak remains in the same position but it takes on a more complicated form. In this experiment there was just one wavelength Cu *K*α_1_ and for a perfect crystal this would be a single peak. As the mirror peak approaches the 2θ_B_ peak the intensity increases.

The diffraction profiles in Fig. 1 ▸ are not easy to explain with conventional theory because the perfect sample has a single peak and a double peak at each Ω incident beam value containing two wavelengths. Bragg’s law would result in two peak positions, one for each wavelength, from which the intensity is dispersed. The profile from the imperfect sample also leads to similar difficulties, in that there is always intensity at 2θ_B_ regardless of the orientation and this is not predicted by Bragg’s law. The description given by Fewster (2014 ▸) refers to the case of a perfect crystal using monochromatic radiation, but it does also imply that all diffraction cannot be described by the simple application of Bragg’s law. There are many publications on dynamical theory covering more than five decades of study of distorted crystals, which has been thoroughly reviewed and discussed by Authier (2001 ▸). The cited publications cover everything from ray tracing and how this can be extended with the description by Takagi (1962 ▸, 1969 ▸), including the breakdown of coherence (Kato, 1976 ▸), to the point where Takagi’s theory becomes invalid at high strain levels and diffuse scattering becomes prominent (Krivoglaz, 1996 ▸). In statistical dynamical theories (Kato, 1976 ▸, for example), it is speculated that the coherence is maintained over certain distances (*e.g*. between defects) and is akin to the mosaic block description. This may well be a good description of the microstructure; however in an attempt to fully explain the experimental evidence it is necessary to consider the diffraction mechanism at a more fundamental level.

## 3. The origin of the intensity   

A crystal can be considered as an ordered array of scattering points, and as the regularity of the array diminishes the crystal becomes imperfect. A three-dimensional array can be viewed such that it appears as many sets of planes. The scattering points can be considered as a unit cell (or repeat entity) composed of atoms or molecules, provided there is a reasonable number of unit cells.

**Figure 3:**
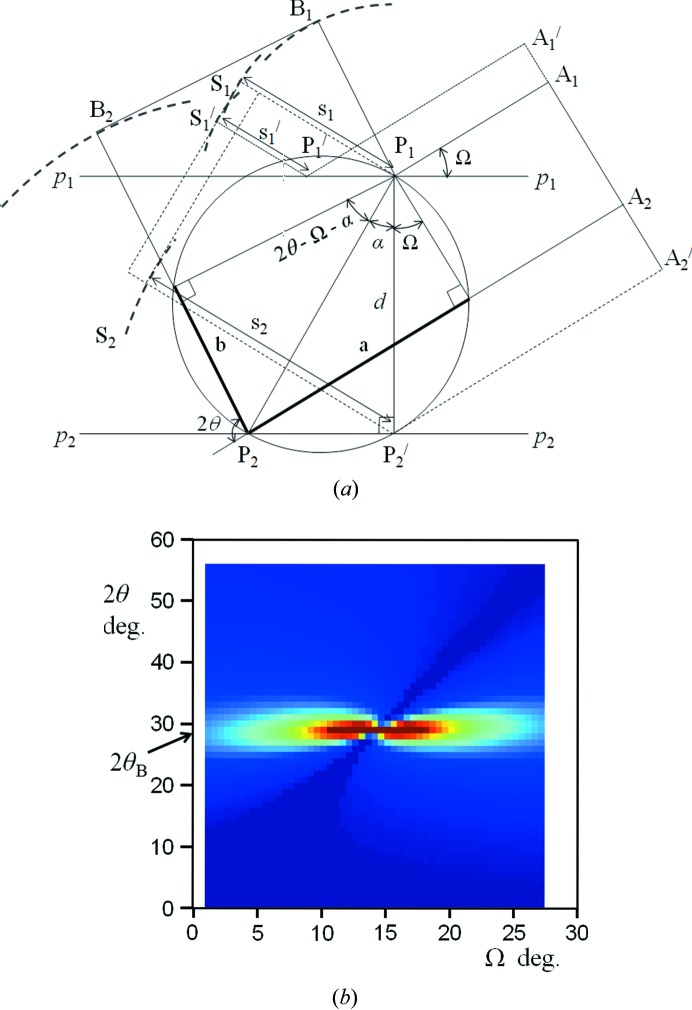
(*a*) *A* represents the incident wavefront, *S* the specular wavefront and *B* the wavefront at the scattering angle 2θ, which is drawn for the direction when the scattering from each plane is in phase. The Bragg equation assumes there is only one peak at the detector and this occurs when both *B* and *S* are coincident, *i.e.* the scattering is a mirror reflection, whereas the new explanation allows *B* and *S* to be separated. (*b*) This is a map of the number of scattering points with a path difference < 10^−4^ nm as a function of Ω and 2θ obtained with steps in α of 10^−5^ rad, plotted on a linear scale from 0 to 2000 scatters (see supporting information). The maximum is at 300 000 for the Bragg condition when all the contributions are in phase.

An incident wave impinging on a plane of scattering points *P* will create spherical waves from each (Fig. 3 ▸ *a*). The maximum amplitude of these waves occurs at a radius *s*_1_ when the incident wave maximum at *A* has travelled from *A*_1_ to *P*_1_ and along *s*_1_. Similarly the radius 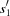 of maximum amplitude occurs when the maximum from 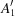 has scattered from 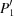 along 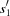 *etc*. These radii of maximum amplitude will merge to form a plane wavefront at *S*_1_ 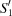, which occurs at the specular condition for each plane of atoms. Another wavefront will form at *S*_2_ from the plane, *p*_2_ *p*_2_, and travel in the same specular direction as *S*_1_, but the maxima are not necessarily coincident with *S*_1_. The combined amplitudes will not create maximum intensity unless the path lengths *A*_1_ *P*_1_ *S*_1_ and 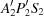 differ by an integer number of wavelengths, which is the Bragg condition. However, the phase combination of the amplitudes scattered in any direction from a single plane will form wavefronts in all directions even if the individual spherical radii are not in perfect phase alignment, the amplitudes are just weaker.

The scattering direction for these weaker amplitudes to be in phase, and therefore give an intensity peak, is determined with reference to Fig. 3 ▸(*a*). The path length *A*_2_ *P*_2_ *B*_2_ will differ by an integer number of wavelengths from *A*_1_ *P*_1_ *B*_1_ at a given incident angle Ω, only when the scattering angle 2θ and angle α (α can take on any value) satisfy the equation
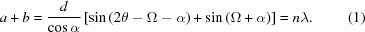
If the points *P*_1_ and *P*_2_ scatter in phase their amplitude contributions will add. The total amplitude is the sum of all the contributions that scatter in a close phase relationship at a specific scattering angle for a given incident angle. From equation (1) we can decide on an acceptable path difference, Δ = |*a* + *b* − *n*λ| and sum the number of α values, for specific Ω and 2θ values, that have a path difference < Δ. These totals are plotted in Fig. 3 ▸(*b*) for *n* = 1 and show that there is an intensity peak at 2θ_B_ for all values of Ω. To maintain phase coherence in large crystals with many planes, the acceptable path difference must be smaller which narrows the width of the peak at 2θ_B_.

## 4. The diffraction from imperfect crystals   

**Figure 4:**
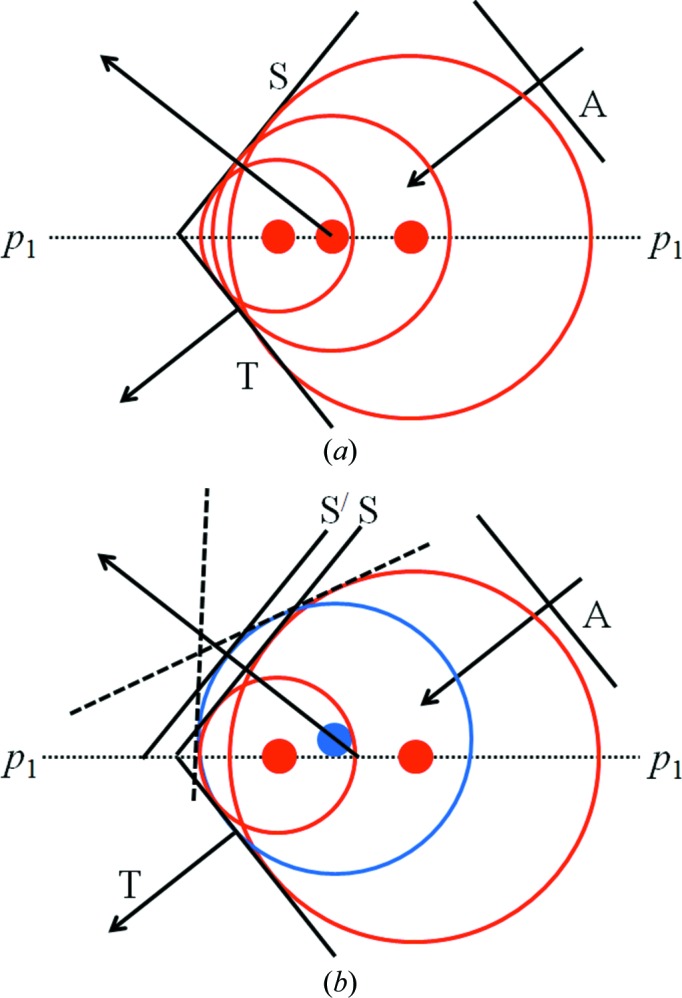
(*a*) The specular wave from a single flat plane of scattering points. (*b*) The specular wave from scattering points that do not lie on a flat plane. *A*, *T* and *S* represent the incident, transmitted and specularly reflected wavefronts, respectively.

The description in Fig. 3 ▸ refers to a perfect crystal when all the scattering points are in the same plane and the maxima of the spherical waves all coincide and form a planar specular wavefront *S* (Fig. 4 ▸ *a*). The crystal planes of an imperfect crystal are not perfectly flat and parallel, due to small defects and strains, and the wavefront is formed from scattering points as shown in Fig. 4 ▸(*b*). The maxima of these waves cannot be brought into coincidence, so the contributions cannot have a perfect phase alignment and the specular peak from such a plane will be weaker and broadened. The angular broadening can be visualized by the dashed wavefronts in Fig. 4 ▸(*b*).

**Figure 5:**
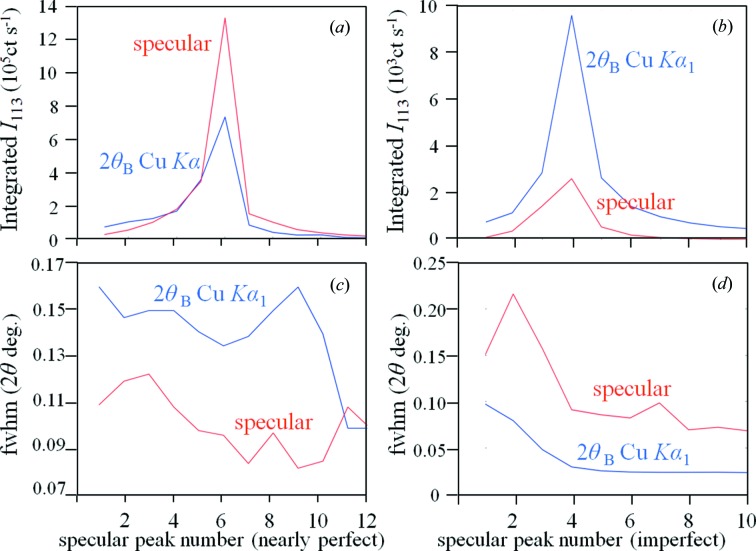
The integrated intensities (*a*) and FWHM (*c*) values for a nearly perfect crystal (Fig. 1 ▸) and (*b*) and (*d*) for the imperfect crystal (Fig. 2 ▸) for those positions where the specular and 2θ_B_ peaks are distinct. The absolute values of the intensities cannot be simply compared between these two crystals, because of the different primary optics, sample-to-detector distance and grazing exit angles of the scattered beam.

A strained crystal has regions of different *d* values giving a range of 2θ_B_ values. This can be seen for the imperfect gallium arsenide crystal in Fig. 2 ▸. Despite the incident beam being monochromatic and of low divergence, the 2θ_B_ peak is ∼3× broader than expected and not a simple shape. The specular peaks are considerably broader and weaker than the 2θ_B_ peaks (Figs. 5 ▸ *b* and 5 ▸ *d*), whereas for the more perfect sample (Figs. 5 ▸ *a* and 5 ▸ *c*), the specular peak is generally more intense than the 2θ_B_ peak, and the peak is narrower.

The suppression of the dynamical effects can be understood by comparing the diffraction from a perfect (Figs. 4 ▸ *a*, 5 ▸ *c*) and an imperfect (Figs. 4 ▸ *b*, 5 ▸ *d*) crystal. Because the specular peak is narrow in the perfect crystal, the crystal plane is close to being flat over a large area, and therefore all the contributions can form a nearly planar wavefront and can simultaneously meet the Bragg condition. This will require dynamical theory to model this scattering. When the planes are bent as in an imperfect crystal only a small range of the broad specular peak can overlap with the 2θ_B_ peak to satisfy the Bragg condition at any single setting. For example the fourth specular peak (Figs. 2 ▸ and 5 ▸ *d*), which is closest to the 2θ_B_ peak, is greater than 3× the width of the 2θ_B_ peak. Therefore the Bragg condition can only be satisfied by a fraction (one third at most) of the crystal at any one setting and hence the dynamical effects are suppressed. The spread in 2θ_B_ from strain reduces the chance of satisfying the Bragg condition further, *i.e.* the relevant *d* spacing for the region being probed corresponds to approximately one third of the peak width (the peak ∼3× broader than expected). In this example therefore the dynamical effects are suppressed by about an order of magnitude.

## 5. Discussion   

All crystals are distorted to an extent because they contain a mixture of dislocations, precipitates and point defects. Curvature of the crystal planes is a natural response to this along with some variation in plane spacing. This alternative explanation suggests that a distorted crystal will place intensity at the Bragg angle without satisfying the Bragg condition, and reduce the impact of dynamical effects. With this new approach the small crystal block model, which is inevitable with Bragg’s interpretation of diffraction, is not necessary here because the interference of imperfectly ordered scatterers accounts for the variation of intensity already.

So it appears that the kinematical theory approximation is appropriate for imperfect crystals. But *F*_*hkl*_ is the total amplitude associated with the crystal plane *hkl*, and will clearly not be localized to the vicinity of the Bragg peak. Consider some possibilities: if the crystal planes are perfectly flat then the intensity scattered by those planes can simultaneously scatter at the specular position with a contribution at the 2θ_B_ position (Fewster, 2014 ▸). This effect is shown in Fig. 5 ▸(*a*) for a relatively perfect crystal, whereas the specular peak is weak compared with the 2θ_B_ peak for the imperfect crystal experiment (Fig. 5 ▸ *b*).

Hence a typical intensity profile measurement obtained with a low-divergence incident beam (ΔΩ) and small acceptance in the scattered beam (Δ2θ) will capture a good proportion of the intensity by scanning along the specular direction with both axes, if the crystal is perfect. If the same experiment is performed on an imperfect crystal then most of the intensity is at 2θ_B_ (Figs. 2 ▸ and 5 ▸ *b*). This is an inconvenience for imperfect single-crystal analysis because the intensity needs to be integrated whilst rotating in Ω to capture the intensity at 2θ = 2Ω and 2θ_B_ to give a reasonable approximation to |*F*_*hkl*_|^2^. This dispersion of the intensity but enhancement at 2θ_B_ is however very convenient in powder diffraction because the whole pattern can be captured from randomly orientated crystals to give a good estimate of |*F*_*hkl*_|^2^, provided a correction for the intensity dispersion is taken into account (Fewster, 2014 ▸).

For the most perfect crystals the 2θ_B_ peak is weak compared with the specular peak and the experimental method described above should lead to good agreement with dynamical theory. As the crystal quality declines as seen in Figs. 1 ▸ and 2 ▸, the 2θ_B_ peak begins to become more dominant, which is not captured by scanning along the specular direction if a small divergence, ΔΩ, and small acceptance in the scattered beam, Δ2θ, are used. This will lead to poor intensity estimates unless the proportion of the specular to 2θ_B_ peak intensities remains constant. In Figs. 5 ▸(*a*) and 5(*b*) this proportion is not constant, the nearly perfect crystal gives a standard deviation over the mean of 0.5 and the imperfect crystal gives 0.7, *i.e.* in this example the reliability of the intensity estimates declines as the perfection decreases, unless more of the dispersed intensity is measured or estimated.

This description accounts for many of the features observed in experiments, without reverting to more complex or unrepresentative structural models. It can be extended to very imperfect crystals provided the scattering points can be represented by the structure factor. For non-periodic structures (amorphous materials) the scattering needs to be considered at the atomic level (Debye, 1915 ▸), and for crystals with only a few unit cells the crystal shape starts to become important. However for most crystals the structure factor is a very convenient description of the scattering from the repeat unit. At present the calculation of the scattering at the atomic level is prohibitive in time with typical size samples. This approach though gives a route to understanding the scattering by X-rays from the most perfect crystals to amorphous materials.

## Supplementary Material

Computer code for generating the diffraction effect given in Fig. 3(b). DOI: 10.1107/S2053273315018975/ae5011sup1.pdf
