# Supplementary material for: What is an ‘ideally imperfect’ crystal? Is kinematical theory appropriate?
Source: Acta Crystallogr A Found Adv. 2016 Jan 1;72(Pt 1):50–4. doi: 10.1107/S2053273315018975 (PMC4689186; doi:10.1107/S2053273315018975)
Supplement: Supplementary file 1 [file a-72-00050-sup1.pdf]

FOUNDATIONS
ADVANCES

Volume 72 (2016)

Supporting information for article:

What is an `ideally imperfect' crystal? Is kinematical theory appropriate?

Paul F. Fewster

The following code is written in Python 2.7 and will reproduce figure 3b. By varying the acceptable phase difference, etc., changes to the enhancement can be seen.

```
import numpy as np
import pylab as p

p.ion()
p.show()

Lambda=0.1540593                    # x-ray wavelength nm
h=np.array([1,1,1])                         # Miller indicies
lattice_parameter=0.543102          # lattice parameter for cubic crystal
Acceptable_phase_difference=1e-4            # acceptable_phase_difference in nm

# evaluate d and TwoTheta_B

d=lattice_parameter/np.sqrt(h[0]**2+h[1]**2+h[2]**2)
TwoTheta_B=2*np.arcsin(Lambda/(2*d))

# define omega range from 1 deg to TwoTheta_B

omega_step=np.radians(0.5)
omega_begin=np.radians(1)
omega_end=TwoTheta_B
omega=np.arange(omega_begin,omega_end,omega_step)

# define TwoTheta range from 1 deg to 2*TwoTheta_B

TwoTheta_step=np.radians(1)
number_of_2T_steps=int(TwoTheta_B/TwoTheta_step)
Down=TwoTheta_B-float(number_of_2T_steps)*TwoTheta_step
Up=2*TwoTheta_B
TwoTheta=np.arange(Down,Up,TwoTheta_step)

# define arrays

Number_in_phase=np.zeros([len(TwoTheta)])
Min_Path_Difference=np.ones([len(TwoTheta),len(omega)])
alpha=np.arange(-np.pi/2.1,np.pi/2.1,1e-5)

for j in range(len(TwoTheta)):
    for i in range(len(omega)):
        Delta=d/np.cos(alpha)*(np.sin(TwoTheta[j]-omega[i]-alpha)+np.sin(omega[i]+alpha))-Lambda
        Delta_small=np.where(np.abs(Delta)<Acceptable_phase_difference)
        Min_Path_Difference[j][i]=len(Delta_small[0])
    print int(np.sum(Min_Path_Difference[j][:]))," in phase at 2Theta =
",np.round(np.degrees(TwoTheta[j]),3)
```

```
# select ranges to plot

Begin=0
Up=2000

# plot and label

p.figure(figsize=(14,12))
alevels=p.pcolor(np.degrees(omega),np.degrees(TwoTheta),Min_Path_Difference,vmin=Begin,vmax
=Up)
p.ylabel('2 Theta')
p.xlabel('omega')
Title='density of near zero phases; 2Theta_B='+str(np.round(np.degrees(TwoTheta_B),3))
p.title(Title)
p.colorbar(alevels)
p.pause(1)

a=raw_input("close all")
p.close('all')
```
